# Efficacy of the Finger Toothbrush as an Alternative Oral Hygiene Method: A Non-randomized Controlled Study

**DOI:** 10.7759/cureus.79427

**Published:** 2025-02-21

**Authors:** Venkatesh V Kulkarni, Mrudula S Chaukaskar, Praveena V Kulkarni, Gourav B Deshmane

**Affiliations:** 1 Oral Pathology and Oral Microbiology, Bharati Vidyapeeth (Deemed To Be University) Dental College and Hospital, Pune, IND; 2 Pharmacology and Therapeutics, Bharati Vidyapeeth (Deemed To Be University) Dental College and Hospital, Pune, IND

**Keywords:** dental plaque, finger toothbrush, manual toothbrush, oral hygiene, plaque index, rural population, traditional oral hygiene habits

## Abstract

Introduction

Oral hygiene practices are essential in the prevention of periodontal diseases and caries, with mechanical plaque removal being a primary factor in reducing their incidence. Rural and underprivileged populations often rely on traditional methods, such as using a finger for teeth cleaning. In this context, the present study used a finger toothbrush as an oral hygiene tool that mimics the traditional habit of cleaning teeth using a finger. This study assessed the plaque removal efficacy of the finger toothbrush by comparing it to the teeth cleaning method using a finger and the manual toothbrush (control).

Methods

This non-randomized controlled trial included 90 participants (45 male participants and 45 female participants aged between 18 and 72 years). They were assigned to three groups: Group A (finger toothbrush), Group B (teeth cleaning using a finger), and Group C (manual toothbrush, control) based on their teeth-cleaning method. After manual scaling and polishing, plaque index scores were assessed on Day 1. Post-intervention plaque index was measured on Day 15 using the Silness and Loe plaque index. Data were analyzed using Chi-square or Fisher's exact test for categorical variables, ANOVA for normally distributed continuous variables, and the Kruskal-Wallis H test for non-normally distributed variables. Wilcoxon's signed rank test was used for intra-group comparisons. A p-value of <0.05 was considered statistically significant.

Results

All inter- and intra-group comparisons were statistically significant (p < 0.05). After 15 days, Group A (finger toothbrush) demonstrated a significant reduction in plaque (median plaque index: 0.62, interquartile range (IQR): 0.69) compared to Group B (teeth cleaning method using a finger), which showed minimal plaque reduction (median plaque index: 1.70, IQR: 0.42). Group C showed the most significant plaque reduction (median plaque index: 0.29, IQR: 0.19). The finger toothbrush was significantly more effective than the teeth cleaning method with a finger in reducing plaque accumulation, though it was not as good as a manual toothbrush.

Conclusion

This study highlighted the importance of alternative oral hygiene methods tailored to populations using the finger for teeth cleaning. The finger toothbrush effectively reduces plaque compared to teeth cleaning using fingers and serves as a valuable transitional tool, promoting better oral hygiene in populations where manual toothbrushes are inaccessible or culturally unaccepted, thereby bridging the gap between traditional and modern oral hygiene practices.

## Introduction

Periodontitis, being an outcome of a lack of awareness of oral hygiene, originates in the oral cavity as a small plaque [1]. Oral hygiene practices are the cornerstone of plaque removal, as they are positively correlated with the incidence of periodontal diseases and caries. Mechanical removal of plaque has a direct correlation with the incidence of caries and periodontal diseases. The use of traditional means of oral hygiene maintenance has a long-recorded history, with widespread usage in rural areas of Africa, South America, and the Indian subcontinent to date [2]. Oral hygiene, the most ignored aspect of oral health in India, is still largely based on traditional practices, especially in rural areas, such as the use of charcoal, mishri, miswak/chew sticks, and fingers for cleaning teeth [3]. Even though toothbrushes are more effective at reducing plaque, traditional methods such as using fingers for teeth cleaning are observed in many parts of India. Statistics on dental health in India reveal that only 51% of the population regularly uses toothbrushes and toothpaste [4]. This indicates that a significant section of the population does not have access to manual toothbrushes. As a result, there is a need for public health interventions to promote proper oral hygiene habits, particularly in rural and lower-income communities.

Oral self-care, including regular tooth brushing and interdental cleaning, is essential for preventing periodontal disease, dental caries, and tooth loss. While traditional oral health promotion focuses on increasing knowledge, a more holistic approach now addresses the underlying social and behavioral factors affecting oral health. Psychological approaches to behavior management, such as the use of reinforcement, goal setting, and the provision of feedback, can improve oral hygiene and oral hygiene-related behaviors [5,6]. The principles of evidence-based medicine state that when giving recommendations regarding the use of oral hygiene devices, one should take into account both individual preferences and dexterities as well as scientific evidence [7]. In this context, the present study used a finger toothbrush as an oral hygiene tool. Its design aligns with the traditional habit of cleaning teeth with a finger, making it a culturally compatible option for improving oral hygiene.

The aim of this study was to evaluate the effectiveness of the finger toothbrush as an alternative oral hygiene tool in populations accustomed to traditional teeth cleaning methods with fingers. The study hypothesized that the use of the finger toothbrush does not significantly improve oral hygiene compared to the finger method, and its efficacy is not comparable to that of a manual toothbrush as a plaque removal tool (null hypothesis). Conversely, it proposed that the finger toothbrush significantly improves oral hygiene compared to the finger method, and its efficacy is comparable to that of a manual toothbrush as a plaque removal tool (alternate hypothesis).

## Materials and methods

Study design

The Institutional Ethics Committee of Bharati Vidyapeeth (Deemed To Be University) Dental College and Hospital, Pune, India, approved this non-randomized controlled trial. We registered the trial in the Clinical Trials Registry of India (CTRI) under the registration number CTRI/2022/10/046701. We conducted the study from 31 October 2022 to 30 November 2022.

Participant recruitment and group allocation

We included a total of 90 participants (45 male participants and 45 female participants) aged between 18 and 72 years based on their method of teeth cleaning. We obtained informed consent from all participants prior to inclusion. Demographic data (age, gender, and occupational status) were recorded for each participant, along with their preferred teeth-cleaning method. To ensure confidentiality, each participant was assigned a unique code number.

**Figure 1:**
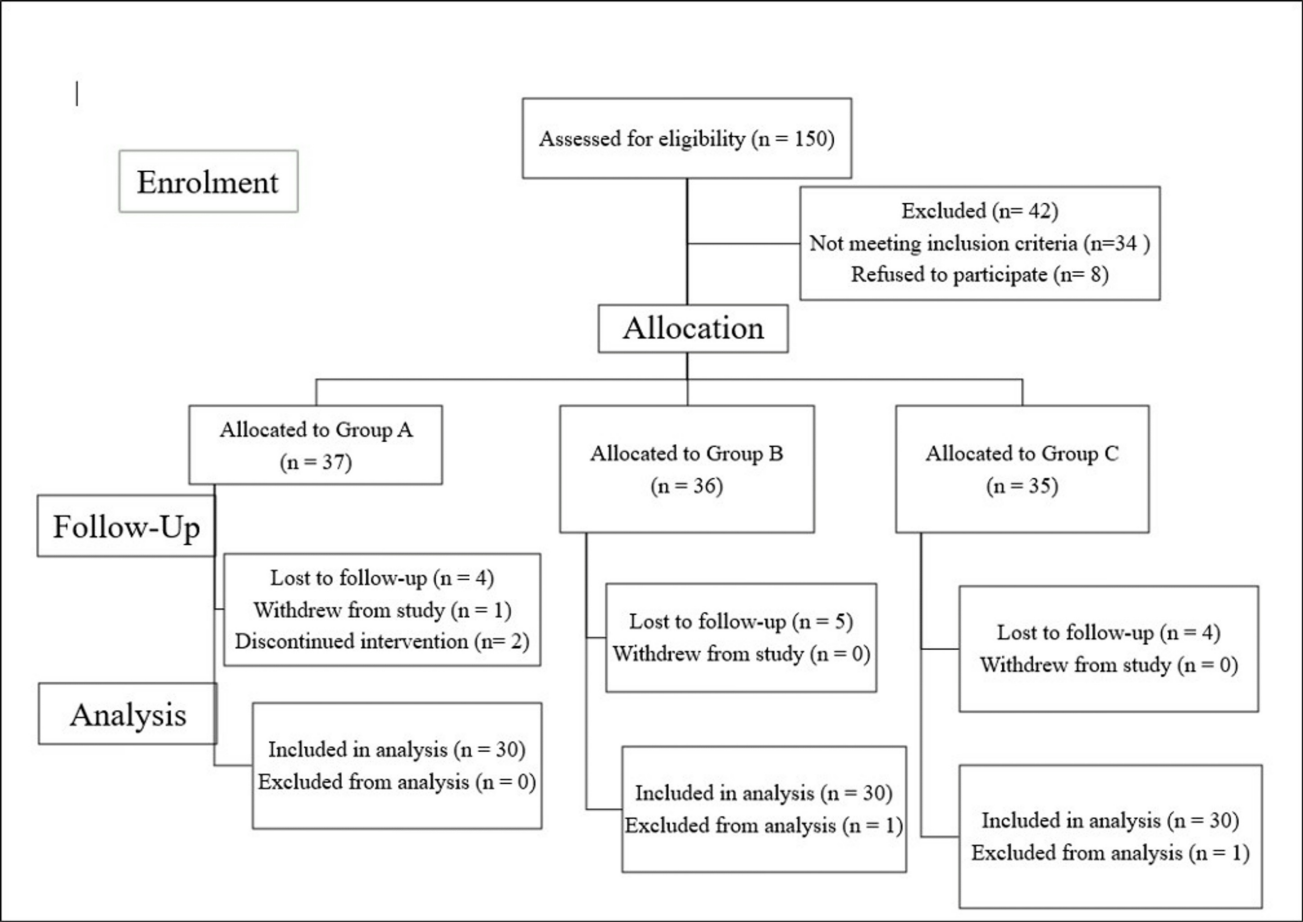
A CONSORT flow diagram of the non-randomized controlled trial CONSORT: Consolidated Standards of Reporting Trials

We assigned 60 finger-tooth-cleaning participants to two groups using convenience sampling: those willing to use a finger toothbrush were included in Group A, and the rest in Group B. Group C, the control group, comprised participants who used manual toothbrushes for teeth cleaning (Figure 1).

Inclusion and exclusion criteria

We included adults with 20 or more natural teeth who had six scorable index teeth (FDI tooth notation: 16, 12, 24, 32, 36, 44) for evaluation. We excluded edentulous individuals, patients on medications, individuals with periodontal disease, and those with restorations, prostheses, or orthodontic appliances on their index teeth.

Sample size estimation formula 

$n(Per Group) = 2\left[\frac{(Z_{\frac{\alpha}{2}} + Z_{\beta}) \sigma}{\Delta}\right] ^2$

Level of significance = 5%, Power = 80%

Where: n = sample size, 𝑍_𝑎/2_ = Critical value from the standard normal distribution (Z-table) corresponding to: α (Type I error rate, usually 0.05); α/2: two-tailed test, dividing α by 2 for each tail; 𝑍_𝛽_ = Critical value from the standard normal distribution (Z-table) corresponding to: β (Type II error rate, usually 0.2 or 0.1); 1 - β: statistical power (e.g., 0.8 or 0.9); σ (Sigma) = Standard deviation of the outcome variable; Δ (delta) = minimum detectable difference (effect size) between groups.

The smallest difference was considered clinically significant.

Intervention

**Figure 2:**
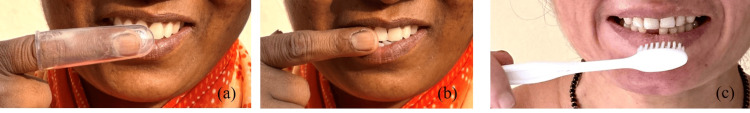
Teeth cleaning method with (a) finger toothbrush, (b) finger, and (c) manual toothbrush Original images from the study.

After manual scaling and polishing, the plaque index scores of each participant were assessed on Day 1. All participants were provided with Colgate toothpaste (Colgate Palmolive Ltd., Mumbai, India) and instructed to brush their teeth twice daily, once in the morning and once at night, for the duration of the 15-day study. Participants in Group A were provided with a finger toothbrush and instructed to use it for the next 15 days (Figure 2). Proper brushing techniques were demonstrated by a trained dental professional, and participants were encouraged to ask questions for clarification. To ensure adherence, participants were contacted through telephonic follow-ups every five days to reinforce the importance of compliance and address any challenges they faced during the intervention.

Four hours prior to the measurement of their post-intervention (Day 15) plaque index, all participants on that day did not engage in any dental hygiene activities.

Outcome measurement

To assess plaque accumulation on the tooth surfaces, the Silness and Loe method was used to record each participant's plaque index. One examiner performed all plaque index assessments; to reduce bias and ensure uniformity in scoring across all assessments, the examiner was blinded to the participants' group assignments. Plaque-staining dye, a disclosing agent, was applied to each participant's teeth to enable clear visualization of plaque deposits and aid in the evaluation. A total of six index teeth, including the maxillary right first molar (Tooth #16), maxillary right lateral incisor (Tooth #12), maxillary left first bicuspid (Tooth #24), mandibular right first bicuspid (Tooth #44), mandibular left lateral incisor (Tooth #32), and mandibular left first molar (Tooth #36), were assessed for plaque on four surfaces (mesiofacial, facial, disto-facial, and lingual).

The amount of plaque on each surface was noted on a four-point scale. A score of 0 indicated no plaque present. Score 1 represented a slight plaque at the gingival margin. Score 2 indicated moderate plaque at the gingival margin, covering a portion of the tooth surface. Score 3 was assigned for heavy plaque accumulation covering a substantial portion of the tooth surface.

Statistical analysis

Independent variables were groups of cases (Group A, Group B, and Group C). The data on categorical variables were shown as n (% of cases, and the data on continuous variables was presented as mean and standard deviation (SD) for normally distributed variables, and non-normally distributed variables, median (min-max) was used. If more than 20% of cells had an expected frequency below five, the chi-square test or Fisher's exact probability test was used to compare the distribution of categorical variables between groups. The ANOVA with post hoc Bonferroni's test for multiple group comparisons was used to statistically compare the means of normally distributed continuous variables between groups. The inter-group statistical comparison of medians of non-normally distributed continuous variables was done using the Kruskal-Wallis H test (non-parametric ANOVA). Wilcoxon's signed rank test was used for intra-group statistical comparisons of the medians of non-normally distributed continuous variables.

Prior to using ANOVA on the study variables, the underlying normality assumption was tested. To better illustrate the statistically significant difference, all results are displayed in both tabular and graphical formats. In the entire study, p-values less than 0.05 were considered statistically significant. The normality assumption was tested for each parameter using Shapiro-Wilk's test.

## Results

Table 1 displays the distribution of participants across the three study groups, with each group consisting of 30 participants. Group C (manual toothbrush) was used as a control group for comparison with the two groups (Group A and Group B). 

**Table 1:** Distribution of the sample size studied across three study groups

Group code	Group description	No. of cases (n)	% of total
Group A	Oral hygiene habit: participant cleaning teeth with a finger toothbrush	30	33.3%
Group B	Oral hygiene habit: participant cleaning teeth with their finger	30	33.3%
Group C	Oral hygiene habit: participant cleaning teeth with a manual toothbrush (control)	30	33.3%
Total		90	100%

Inter-group comparison of mean age

The mean ± SD age of participants in Group A, Group B, and Group C was 42.8 ± 15.3 years, 44.2 ± 13.5 years, and 42.5 ± 14.9 years, respectively. The age range for Group A was 18-71 years, for Group B was 18-70 years, and for Group C was 18-72 years. There was no significant difference in the distribution of mean age between the groups (P-value > 0.05).

Inter-group comparison of gender

In Group A, 12 (40%) participants were male and 18 (60%) were female. In Group B, 14 (46.7%) were male participants and 16 (53.3%) were female participants. In Group C, 17 (56.7%) were male participants and 13 (43.3%) were female participants. The gender distribution did not differ significantly between the groups (P-value > 0.05).

Inter-group comparison of occupation

In Group A, 10 (33.3%) were housewives, four (13.3%) were farmers, nine (30%) were laborers, five (16.7%) held various occupations (mechanic, driver, engineer, teacher), and 2 (6.7%) were students. In Group B, seven (23.3%) were housewives, 10 (33.3%) were farmers, eight (26.7%) were laborers, two (6.7%) held various occupations, and one (3.3%) was a student. In Group C, seven (23.3%) were housewives, two (6.7%) were farmers, nine (30%) were laborers, nine (30%) held various occupations, and three (10%) were students. The distribution of occupational status did not differ significantly between the groups (P-value > 0.05).

All demographic characteristics showed no statistically significant differences between the groups (P-values > 0.05), indicating the groups were well-balanced for gender, age, and occupation (Table 2).

**Table 2:** Demographic characteristics of participants across groups P-value<0.05 (statistically significant); NS: statistically non-significant

Demographic characteristics	Group A (n=30)	Group B (n=30)	Group C (n=30)	P-value
Age				
Age range in years	18–71	18–70	18–72	0.931^NS^
Mean ± SD	42.8 ± 15.3	44.2 ± 13.5	42.5 ± 14.9	
Gender				
Male	12 (40%)	14 (46.7%)	17 (56.7%)	0.421^NS^
Female	18 (60%)	16 (53.3%)	13 (43.3%)	
Occupation				
Housewife	10 (33.3%)	7 (23.3%)	7 (23.3%)	0.743^NS^
Farmer	4 (13.3%)	10 (33.3%)	2 (6.7%)	0.071^NS^
Labourer	9 (30%)	8 (26.7%)	9 (30%)	1.000^NS^
Mechanic/Driver/Engineer/Teacher	5 (16.7%)	2 (6.7%)	9 (30%)	0.076^NS^
Student	2 (6.7%)	1 (3.3%)	3 (10%)	0.783^NS^

In comparison to the finger method of teeth cleaning, Group A (finger toothbrush) showed a median plaque index of 0.62 (interquartile range (IQR): 0.69) after the intervention. This indicates that the finger toothbrush effectively provides a meaningful hygiene improvement for those previously using their finger for teeth cleaning. Group B (finger method of teeth cleaning) showed the least effectiveness in plaque reduction, with a plaque index median of 1.70 (IQR: 0.42) after 15 days. This underscores the minimal plaque-removing capacity of the finger method alone, indicating its inadequacy in preventing dental plaque accumulation. The post-intervention plaque index was lowest in Group C (manual toothbrush-control group), with a median of 0.29 (IQR: 0.19). This confirms that, among the three groups, the manual toothbrush is the most effective tool for plaque removal (Table 3).

**Table 3:** Inter-group and intra-group comparisons of the median plaque index on Day 1 and after 15 days Values are median and IQR. The P-value (inter-group) was calculated by the Kruskal-Wallis H test (non-parametric ANOVA), and the P-value (intra-group) was calculated by Wilcoxon’s signed rank test. A P-value < 0.05 was considered to be statistically significant. P-value<0.05, *P-value<0.01, ***P-value<0.001, NS: statistically non-significant. A single hyphen (-) indicates that the data is not applicable.

	Group A (n = 30)		Group B (n = 30)		Group C (n = 30)		P-value (Inter-Group)		
Plaque index (PI)	Median	IQR	Median	IQR	Median	IQR	A vs B	A vs C	B vs C
Day 1	0.00	0.00	0.00	0.00	0.00	0.00	0.999^NS^	0.999^NS^	0.999^NS^
After 15 days	0.62	0.69	1.70	0.42	0.29	0.19	0.001^***^	0.001^***^	0.001^***^
P-value (Intra-Group) Day 1 vs. Day 15	0.001^***^		0.001^***^		0.001^***^		-		

**Figure 3:**
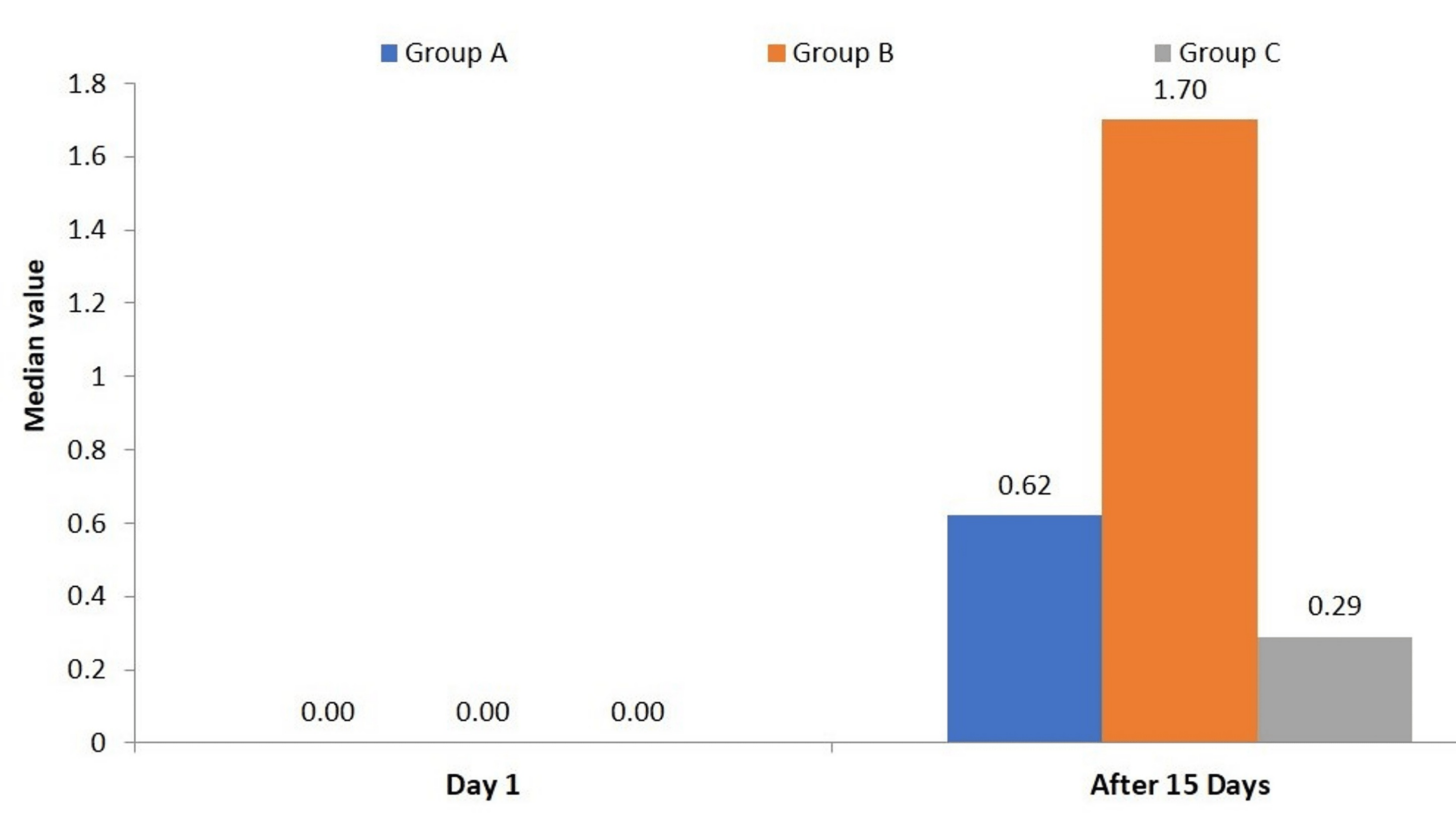
Inter-group distribution of the median plaque index on Day 1 and after 15 days

The inter-group comparison of the median plaque index values on day one and after 15 days highlighted distinct differences in plaque removal efficiency among the three groups. In Group A, the median (IQR) plaque index on Day 1 and after 15 days was 0.00 (0.00) and 0.62 (0.69), respectively. In Group B, the median (IQR) plaque index on Day 1 and after 15 days was 0.00 (0.00) and 1.70 (0.42), respectively. In Group C, the median (IQR) plaque index on Day 1 and after 15 days was 0.00 (0.00) and 0.29 (0.19), respectively. Group B has a significantly higher median plaque index (distribution among the cases analyzed after 15 days than Groups A and C (P-value<0.05 for both). The median plaque index distribution among the cases under study is higher in Group A than in Group C after 15 days (P-value<0.05) (Figure 3).

## Discussion

This study is the first of its kind to evaluate the effectiveness of the finger toothbrush in reducing plaque accumulation compared to both the traditional finger method of teeth cleaning and the manual toothbrush.

Inter-group comparisons revealed statistically significant differences in plaque reduction between all three groups (p < 0.001). Control Group C, with participants using a manual toothbrush, demonstrated the greatest plaque reduction, as evidenced by the lowest post-intervention plaque index (median: 0.29, IQR: 0.19). This aligns with established evidence that manual toothbrushes are the benchmark for mechanical plaque removal.

The teeth cleaning method with a finger (Group B) proved to be the least effective in controlling plaque, as evidenced by its significantly higher post-intervention plaque index of 1.70 (IQR: 0.42). This finding highlighted the importance of public health initiatives aimed at encouraging the adoption of more effective oral hygiene tools.

Conversely, the plaque control achieved by finger toothbrush (Group A) and manual toothbrush (Group C) was superior to that of the finger method of teeth cleaning (Group B), with manual toothbrush (Group C) showing marginally better performance than finger toothbrush (Group A) after the 15-day period. The post-intervention plaque index for the finger toothbrush (Group A) was 0.62 (IQR: 0.69), which is a significant decrease from the teeth cleaning method with the finger but not as good as the manual toothbrush (Group C). This indicated that the finger toothbrush can serve as an effective transitional tool for populations where traditional practices prevail or with limited access to toothbrushes. Its design appears to bridge traditional and modern oral hygiene practices.

Bhat et al. and Pulikkotil et al. underscored the influence of cultural practices and limited access to dental care resources on oral health. They highlighted the link between inadequate oral hygiene and the high prevalence of periodontal diseases in rural populations of India [8, 9]. However, the present study explored the finger toothbrush as a practical solution for improving oral hygiene in populations where traditional practices prevail or with limited access to toothbrushes.

Shah et al. found that traditional oral hygiene methods, such as charcoal or tobacco-based tooth powders, the bark of neem or mango trees, or simply the water and finger method, were inferior in plaque control as compared to toothpaste and toothbrush use. The manual toothbrush, as expected, remained the most effective [10]. This is consistent with the results of the present study, where participants using only their fingers for teeth cleaning (Group B) showed a limited capacity for plaque control. In contrast, the finger toothbrush, designed to be a traditional, similar transition from the finger method, showed improved efficacy, as seen in the significant reduction in plaque index among participants in Group A. This suggests that the finger toothbrush not only aligns with cultural practices but also provides improved oral hygiene without introducing an unfamiliar tool such as a manual toothbrush.

The study on the impact of manual toothbrush design by Axe et al. (2023) highlighted the significant role of toothbrush design in enhancing plaque removal efficacy, which can influence user compliance and outcomes in oral hygiene [11]. Similarly, the present study recognized the need for tools that can influence user compliance and outcomes in oral hygiene by introducing a finger toothbrush as a culturally compatible tool for populations accustomed to teeth cleaning with a finger to promote optimal oral hygiene.

Neelamana et al.'s findings align with the present study's concern and showed that a greater percentage of the study population used only a finger for cleaning their teeth, which led to poor oral hygiene [12]. However, the finger toothbrush intervention in the present study showed improvement in oral hygiene outcomes in such populations in India.

In this study, the finger toothbrush demonstrated substantial improvement over the finger method in plaque removal but did not achieve the effectiveness of the manual toothbrush. This finding is consistent with previous studies, which underscore the manual toothbrush as the most effective tool for plaque removal [13, 14].

The present study builds on its earlier research on the finger toothbrush by contrasting its effectiveness with that of manual toothbrush (control group). To evaluate the efficacy of the finger toothbrush, the present study utilized the plaque index, while previous research applied the Oral Hygiene Index-Simplified (OHI-S) [15]. Both studies reflect a shared objective of improving oral hygiene among populations accustomed to the traditional finger method of teeth cleaning, with the finger toothbrush as an alternative oral hygiene method. However, the current study emphasized the significance of culturally compatible oral hygiene tools, positioning the finger toothbrush as a bridge between traditional practices and modern oral hygiene solutions for communities resistant to adopting manual toothbrushes.

The results of this study emphasized the potential of the finger toothbrush as an effective tool for improving oral hygiene in populations where manual toothbrushes are either culturally unacceptable or inaccessible. However, the study has a few limitations that could impact its generalizability. Firstly, the use of convenience sampling instead of random sampling. Random sampling may have led to challenges in participant recruitment, as this study required individuals who traditionally use the finger for teeth cleaning, a practice more prevalent in rural and lower-income populations. Conducting randomized sampling in communities with limited healthcare access and varying literacy levels could pose ethical and logistical difficulties. Convenience sampling made the study feasible and ethical while respecting participants' comfort and availability. Secondly, the study's relatively short follow-up period of 15 days restricted the ability to assess the long-term efficacy and adherence to the finger toothbrush. Furthermore, the study focused on a specific demographic, individuals accustomed to traditional teeth-cleaning practice using a finger. Future research with longer follow-up periods and randomized controlled trials could further validate these findings and explore the long-term impact of the finger toothbrush on oral health.

## Conclusions

This study contributes to the growing evidence supporting the need for alternative oral hygiene methods tailored to the habits of populations that use the finger for teeth cleaning. The finger toothbrush significantly reduces plaque compared to the teeth cleaning method with a finger. It is particularly valuable for public health initiatives in rural areas, promoting better oral hygiene and reducing the risk of plaque-related diseases. While a manual toothbrush remains the superior choice for plaque removal, the finger toothbrush is best regarded as a transitional tool to improve oral hygiene in areas where manual toothbrushes may not be accepted or accessible.
